# Assessment of *Toxoplasma gondii* lytic cycle and the impact of a gene deletion using 3D label-free optical diffraction holotomography

**DOI:** 10.3389/fcimb.2023.1237594

**Published:** 2023-08-02

**Authors:** Zisis Koutsogiannis, John G. M. Mina, Rakesh Suman, Paul William Denny

**Affiliations:** ^1^ Department of Biosciences, Lower Mountjoy, University of Durham, Durham, United Kingdom; ^2^ Tomocube, Daejeon, Republic of Korea

**Keywords:** *Toxoplasma gondii*, apicomplexa, optical diffraction holotomography, 3D imaging, label-free imaging

## Abstract

*Toxoplasma gondii* is a widespread single-celled intracellular eukaryotic apicomplexan protozoan parasite primarily associated with mammalian foetal impairment and miscarriage, including in humans. Is estimated that approximately one third of the human population worldwide is infected by this parasite. Here we used cutting-edge, label-free 3D quantitative optical diffraction holotomography to capture and evaluate the *Toxoplasma* lytic cycle (invasion, proliferation and egress) in real-time based on the refractive index distribution. In addition, we used this technology to analyse an engineered CRISPR-Cas9 *Toxoplasma* mutant to reveal differences in cellular physical properties when compared to the parental line. Collectively, these data support the use of holotomography as a powerful tool for the study of protozoan parasites and their interactions with their host cells.

## 1 Introduction 

*Toxoplasma gondii* (from the Greek toxon meaning bow), one of the world’s most successful parasites, is in the class Coccidia of the phylum of Apicomplexa. It can infect almost all warm-blooded animals including humans, livestock and domesticated animals (Dubey, 2021). Following an acute phase (tachyzoites form), the parasite establishes a long lasting latent infection (bradyzoite form) in various tissues including skeletal muscles, the brain and the retina (Channon et al., 2000). During tachyzoite invasion a membrane-bound non-fusogenic compartment known as the Parasitophorous Vacuole (PV) is formed, and is localized close to host Golgi, mitochondria and ER (Sinai et al., 1997; Romano et al., 2013). Sexual development occurs only within enterocytes of the feline intestine with diploid unsporulated oocysts shed in faeces and undergoing meiosis to yield infective sporozoites (Dubey and Frenkel, 1972; Dubey, 1977; Dubey, 1997; Sinai et al., 1997). This is one source of infection, however *Toxoplasma* is also a major foodborne pathogen, largely *via* bradyzoite infected meat, with over 1 million cases per annum in Europe (WHO, 2015). In total, approximately 30% of the world population have been reported to have a chronic infection (Flegr et al., 2014). Whilst the majority of infected individuals do not develop acute symptoms, instead harbouring the slow growing bradyzoite form, the immunocompromised are at a high risk of developing a severe tachyzoite mediated disease such as toxoplasmic encephalitis. Additionally, congenital toxoplasmosis can lead to microcephaly seizures, intellectual disability, hydrocephalus or miscarriage (McLeod et al., 2000).

Microscopy is key to understanding the interaction of *Toxoplasma*, and any other pathogen, with its host, thereby shedding light on the pathology of disease and means of mitigating this. Conventional light microscopy techniques are incapable of measuring physical properties, whilst fluorescence techniques such as scanning confocal microscopy require the use of fluorescent tagging probes or proteins which have numerous limitations in analysing dynamic alterations in cells mainly due to photobleaching and phototoxicity (Kim et al., 2021). However, recent advances in label-free imaging technologies have made it possible to study biological systems at a high spatial resolution, in the *Toxoplasma* field infrared microspectroscopy has recently been employed to analyse chemical changes induced by infection of human brain microvascular endothelial cells (Elsheikha et al., 2022), and the utility of optical diffraction holotomography (ODH) has also been demonstrated (Firdaus et al., 2020). ODH exploits the intrinsic optical properties of a sample and allows the direct calculation of the optical phase delay introduced by refractive index (RI) alterations in live biological samples over relatively long time spans (Kim et al., 2021). Calculating the phase shift in a hologram taken 360° around the sample allows quality improvement and the facilitates label-free, high resolution 3D imaging and quantitative imaging to render precise measurements of cell and organellar volume, surface area and dry mass (Kim et al., 2021).

In this study, to take the observations previously made with *Toxoplasma* (Firdaus et al., 2020) further, we used ODH to assess and analyse the *Toxoplasma* lytic cycle - invasion, proliferation and egress. Moreover, we demonstrated the utility of this technology to analyse genetically engineered *Toxoplasma* mutant cells for associated alterations in volume, surface area and dry mass.

## 2 Materials and methods

All materials were obtained from Thermo Fisher Scientific unless otherwise stated.

### 2.1 *Toxoplasma gondii* strains and host cells

Chinese Hamster Ovary (CHO-K1 CHO-K1 - ATCC^®^ CCL-61) cell lines were used to cultivate *T. gondii* tachyzoites and cultivated in Dulbecco’s Modified Eagle Medium supplemented with 10% foetal bovine serum (FBS), 2 mM L-Glutamine, 1% penicillin/streptomycin, 1x non-essential amino-acids and maintained at 37°C and 5% CO_2_. Tachyzoites were harvested 4 to 5 days after host cell infection and their viability (≥95%) was determined by trypan blue before downstream experiments. *Toxoplasma gondii* strains used in the study: RH.Δku80 (Pieperhoff et al., 2015) as the parental control and RH.Δku80.ΔCerS1 which lacks the lipid biosynthetic enzyme ceramide synthase and demonstrates reduced *in vitro* proliferation (Koutsogiannis et al., 2022).

### 2.2 Optical diffraction holotomography and experimental set up

3D Quantitative images of *Toxoplasma* were produced by using a commercial holotomographic microscope (HT-2H, Tomocube Inc.) that employs ODT using two UPLSAP 60X NA 1.2) water dipping lenses (Olympus, Tokyo, Japan). Full details of the optical configuration have been previously described (Firdaus et al., 2020). CHO-K1 cells were seeded in growth media on specialized glass bottom TomoDishes for 65% to 70% confluency and left overnight to attach properly. 10^4^ RH.Δku80 and RH.Δku80.ΔCerS1 were used to infect host cells which were then monitored over time. The microenvironment in the microscope chamber was kept stable at 37°C, 5% CO_2_. No dyes or other staining agents were used.

### 2.3 3D reconstruction and data analysis

Images were processed and analysed using Tomostudio, Tomocube’s analysis software. ImageJ was also used to analyse microscopic imaging data acquired. Numeric data were analysed with Prism and are expressed as mean ± SD and the significance of differences found between groups determined using the independent Student’s t test as indicated in figure legends. ^*^p<0.05; ^**^p<0.01

## 3 Results

**Figure 1:**
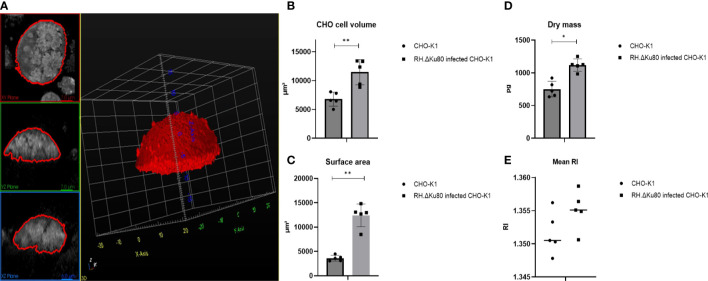
RH.ΔKu80 infected CHO-K1 host cell segmentation 72 hrs post infection **(A)** and physical properties analyses including cell volume **(B)**; surface area **(C)**; dry mass **(D)**; mean RI **(E)**. Values are expressed as mean ± SD, (n=5; ≥ 3 and ¾ 5 PVs), P value significance thresholds were set at: ^*^p<0.05; ^**^p<0.01.

In an initial study, designed to establish the approach to be taken, alterations in the host cell physical properties were monitored during *Toxoplasma* infection using ODH. After segmentation and analyses of individual host cell (n=5) properties (**Figure 1A**) it was observed that, compared to non-infected controls, cell volume, dry mass and surface area of infected cells increased dramatically prior to parasite egress and host cell membrane rupture (**Figures 1B–D**). More precisely, *Toxoplasma* infected CHO cells were found to almost double their dry mass and volume from 750.76 ± 119.33 pg to 1119.45 ± 94.90 pg; and 6776.60 ± 1220.75 μm^3^ to 11461.97 ± 2190.42 μm^3^ respectively. In parallel, surface area was found to be three times larger in infected host cells, from 3612.34 ± 591.11 μm^3^ to 12815.88 ± 2495.05 μm^3^ (**Figure 1C**). RI was not found to have a significant difference between two groups (**Figure 1E**). For this comparison fully loaded CHO cells with more than three and less than five PVs were selected as shown in **Figure 1A**. These data match well with those reported using the same system (Firdaus et al., 2020) and gave us confidence in the approach taken.

**Figure 2:**
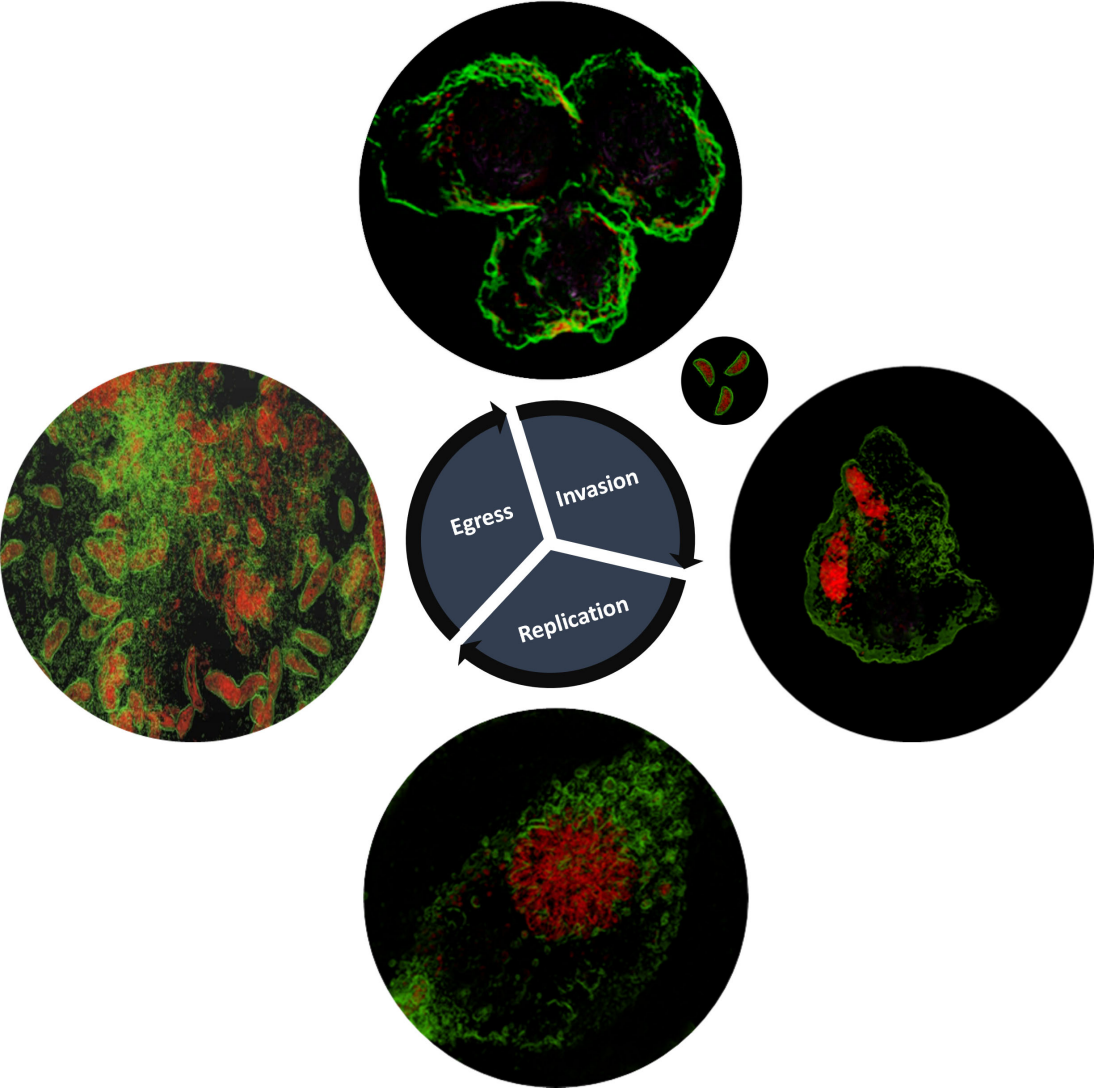
*Toxoplasma gondii* RH.Δku80 lytic life cycle as depicted by holotomography, including invasion into the CHO-K1 host cell, replication and, finally, the host cell membrane rapture and parasite egress. The images shown are representative of multiple experiments with invasion observed and captured 15 minutes post-infection, replication 48 hours post-infection (including rosette formation) and egress and host cell lysis 72 hours post-infection. *Toxoplasma* depicted in red and host cell membranes in green.

During invasion (**Figure 2**), acute phase tachyzoites assemble a moving junction in host’s plasma membrane that creates a ring around the parasite at the point of entry. Micronemal and rhoptry proteins are later secreted (Alexander et al., 2005; Besteiro et al., 2009) that interact with host cell membrane and enable invasion. Later, proteins in the basal portion of the rhoptry are also secreted and mediate alterations in the host cell response and formation of the PV in which the protozoa will proliferate. Using the ODH technology, the characteristic features of the *Toxoplasma* acute lytic cycle could be captured in great detail in living cells (**Figure 2**): parasite invasion: rosette formation (which is the product of successive endodyogeny events where two offspring organisms are assembled within the primary mother cell): and egress and host cell lysis. Furthermore, this technique also allowed the imaging of the PV membrane in live infected host cells (**Figure S1**). Together, these observations showed that ODH technology has application in furthering the understanding of *Toxoplasma* pathobiology in living systems.

**Figure 3:**
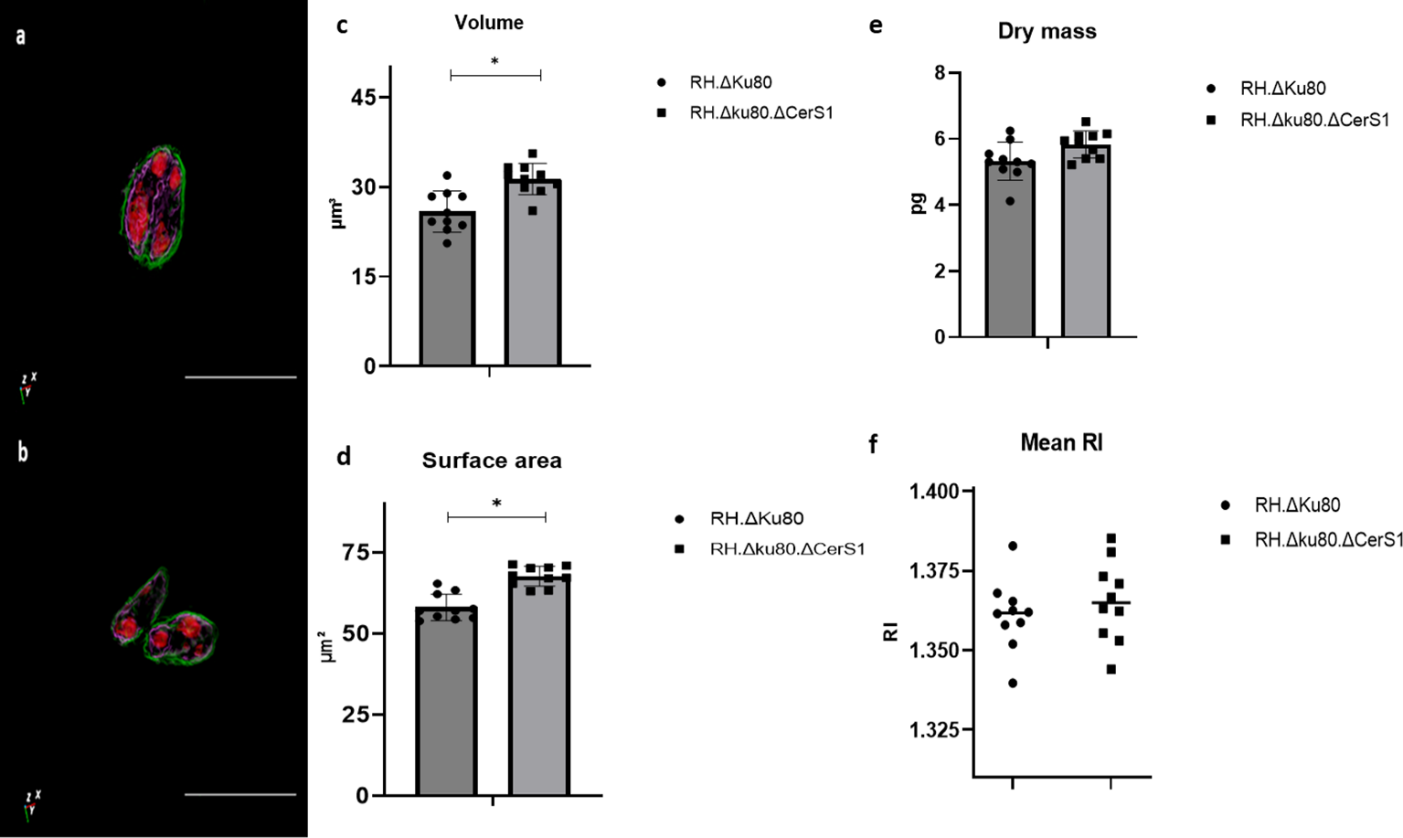
ηolotomography of individual extracellular *Toxoplasma* parasites RH.Δku80 **(A)** and RH.Δku80.ΔCerS1 **(B)** as reconstructed by ODH. *Toxoplasma* plasma membrane is depicted in green and, based on localisation and structure, the internal membrane complex in purple and the conoid and nucleus in red. Scale bar 10μm. RH.Δku80 and RH.Δku80.ΔCerS1 physical properties including volume **(C)**; surface area **(D)**; dry mass **(E)** and mean RI **(F)**. Values are expressed as mean ± SD, (n=5), P value significance thresholds were set at: ^*^p<0.05.

Furthermore, it was shown that ODH 3D label-free and quantitative live-cell imaging enabled the measurement of the physical parameters of two different *Toxoplasma* lines, parental and a mutant lacking the lipid biosynthetic enzyme, ceramide synthase (**Figure 3A, B**). This comparison was based on RI tomogramic profile of 10 tachyzoites of each *Toxoplasma* strain respectively. The mean volume of the RH.Δku80 tachyzoites was measured as 25.87 ± 344 μm^3^ with those of RH.Δku80.ΔCerS1 mutant slightly, but significantly, bigger at 31.31 ± 2.61 μm^3^ (**Figure 3C**). A similarly small but significant increase was observed in the surface area when compared to the parental RH.Δku80, 67.74 ± 3.05 μm^2^ verses 58.16 ± 4.08 μm^2^ (**Figure 3D**). A small but not statically significant difference was observed for dry mass, 5.83 ± 0.40 pg verses 5.32 ± 0.57 pg (**Figure 3E**). No difference was observed in the RI (**Figure 3F**).

## 4 Discussion

To conclude, in this work we evaluated the lytic cycle of *Toxoplasma* based on its unique RI and ODH on a HT-2 microscope (Tomocube) and analysed a characterised mutant, RH.Δku80.ΔCerS1. The results showed that this high content, high-resolution imaging technique was capable of providing valuable real-time information, an approach applicable to not just *Toxoplasma* but also to other intracellular pathogens to increase understanding and, consequently, accelerate research.

More specifically, based on ODH measurements and analyses, RH.Δku80.ΔCerS1 parasites were found to be larger than the parental RH.Δku80. Ceramide synthases are key enzymes in *de novo* sphingolipid biosynthesis that catalyse the formation of ceramide, the basic building block of all sphingolipids and associated with cell signalling pathways including apoptosis, differentiation, inflammation and proliferation (Hannun and Obeid, 2002; Spiegel and Milstien, 2002; Lavieu et al., 2006; Snider et al., 2010). Loss of the gene (TGGT1_316450) that encodes this enzyme *Toxoplasma gondii* (RH.Δku80.ΔCerS1) has a mild impact on parasites fitness (Koutsogiannis et al., 2022). Using the approach described here, this phenotype could be explained by the observed alterations in physical properties, with a larger and differently shaped parasite indicative of major alterations in the cell surface and consequently potential defects in invasion, replication and egress.

In comparison to other imaging techniques, ODH does not require the use of labelling agents or invasive approaches such as fixation which could introduce artefacts which prevent true physiological understanding. Furthermore, as a rapid live imaging technique the tracking of alterations in the physical properties of cells in real time is made possible. Currently, only individual cells can be tracked and analysed separately after segmentation. However, with improved machine learning approaches millions of cells, including pathogens, could be analysed in real time providing valuable information regarding pathogenicity, drug responses and pathobiological effects. Indeed, high content imaging-based techniques have emerged as essential tools in many areas of scientific research, including elucidating the pathophysiology of infectious disease (Firdaus et al., 2020; Elsheikha et al., 2022). Their power lies in the amount of quantitative information that can be derived in real time without labelling. These data can be subsequently associated with biological function, proving the axiom: “seeing is believing”.

## Data availability statement

The original contributions presented in the study are included in the article/**Supplementary Material**. Further inquiries can be directed to the corresponding author.

## Author contributions

ZK was, with the support of JM and RS, responsible for the analyses, interpretation and presentation of the data. PD was the project lead and grant awardee. ZK and PD were responsible for the writing and editing of the manuscript. All authors contributed to the article and approved the submitted version.
