# Supplementary material for: Assessment of Toxoplasma gondii lytic cycle and the impact of a gene deletion using 3D label-free optical diffraction holotomography
Source: Front Cell Infect Microbiol. 2023 Aug 2;13:1237594. doi: 10.3389/fcimb.2023.1237594 (PMC10433743; doi:10.3389/fcimb.2023.1237594)
Supplement: Supplementary file 1 [file DataSheet_1.pdf]

Supplementary Material

S1 Figure. *Toxoplasma gondii* RH.Δku80 parasitophorous vacuole (PV) formation 12 hours post-infection. Three PVs (red arrows in c.) and two host nuclear structures (white arrows in c.) were evident in this dividing host cell. a. Host cell membranes in green; b. *Toxoplasma* in red; c. PV membranes / host nuclear structures in purple; d. merge of a.-c. Scale bars represent 10 μm.
