# Supplementary figures and images for: Assessment of Toxoplasma gondii lytic cycle and the impact of a gene deletion using 3D label-free optical diffraction holotomography
Source: Front Cell Infect Microbiol. 2023 Aug 2;13:1237594. doi: 10.3389/fcimb.2023.1237594 (PMC10433743; doi:10.3389/fcimb.2023.1237594)

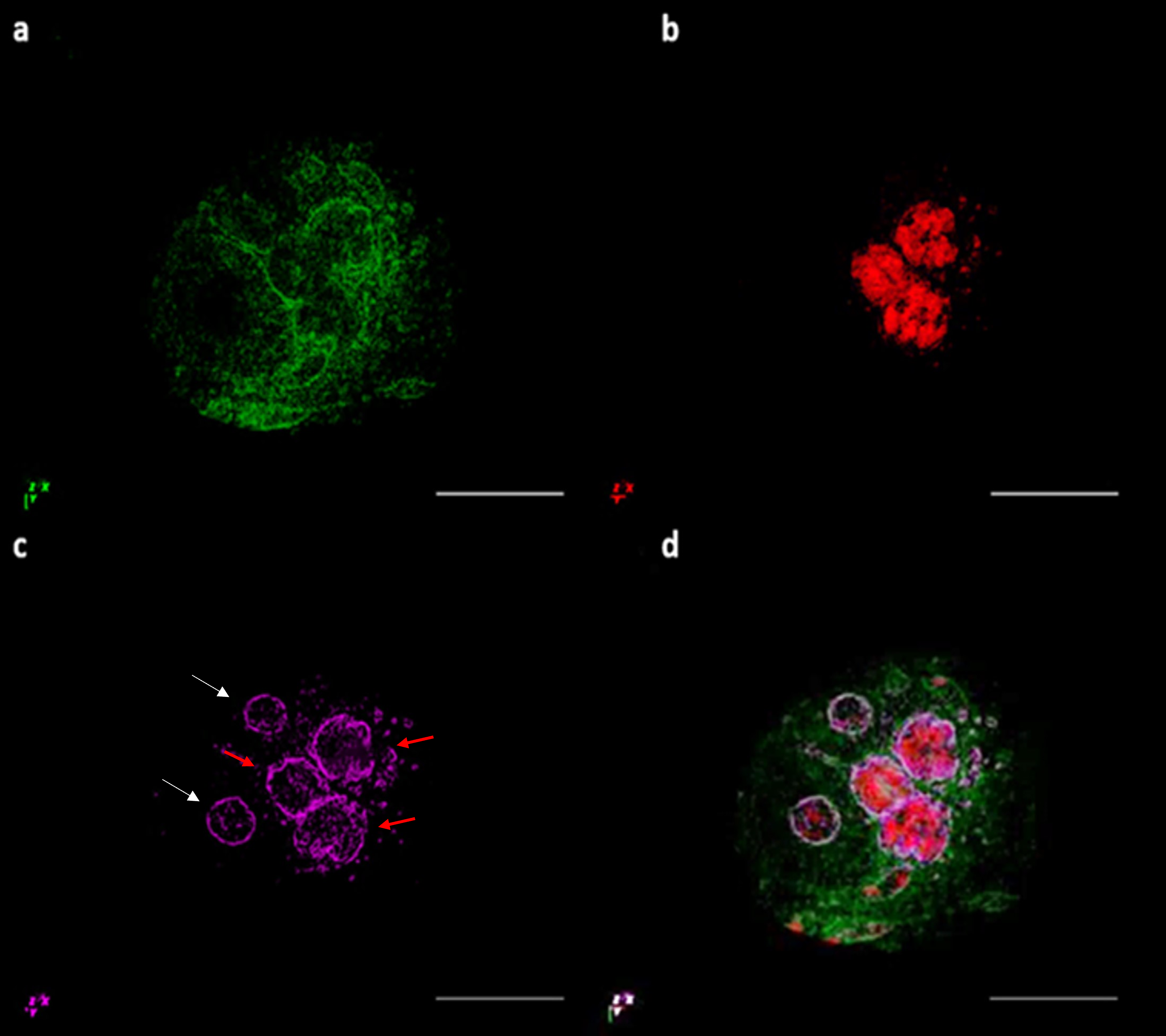

Supplement: Supplementary file 2 [file Image_1.tif]
